# Assessment of phenolic profile and antioxidant power of five pistachio (*Pistacia vera*) cultivars collected from four geographical regions of Iran

**Published:** 2018

**Authors:** Seyedeh Faezeh Taghizadeh, Gholamhossein Davarynejad, Javad Asili, Seyed Hossein Nemati, Gholamreza Karimi

**Affiliations:** 1 *Ferdowsi University of Mashhad, Faculty of Agriculture, Department of Horticulture and Landscape Engineering, Mashhad, Iran*; 2 *Department of Pharmacognosy, Faculty of Pharmacy, Mashhad University of Medical Sciences, Mashhad, Iran*; 3 *Pharmaceutical Research Center, Faculty of Pharmacy, Mashhad University of Medical Sciences, Mashhad, Iran*

**Keywords:** Antioxidant activity, DPPH, Ferric reducing, Pistacia vera

## Abstract

**Objective:**

In this study, the levels and antioxidant activities of some secondary metabolites isolated from five pistachio (*Pistacia vera*) cultivars collected from four different geographical regions of Iran, were studied.

**Materials and Methods:**

Total phenolic compounds levels were determined by Folin-Ciocalteu method. Total flavonoid content was determined as AlCl_3_ complex and expressed as mg of quercetin equivalents (QE)/g dry extract and total proantocyanidins content was expressed as mg of catechin equivalents (CA)/g dry extract. In order to evaluated the antioxidant activity of the compounds, DPPH and FRAP assays were used.

**Results:**

The highest level of total phenols (156.42 mg GA/g DE), total flavonoids (130.94 mg QE/g DE) and total proantocyanidins (152.816 mg CA/g DE) were obtained in Akbari cultivar from Rafsanjan, followed by Badami-e-sefid and Ahmad aghaei. The lowest amount of total phenolic content (TPC), total flavonoid content (TFC) and total proanthocyanidin content (TPrAC) were found in Badami-e-sefid from Feizabad (128.140 mg GA/g DE, 93.176 mg QE/g DE and 118.870 mg CA/g DE, respectively). Also, a positive correlation (r^2^=0.9834) was found between antioxidant activity and total phenolic compounds.

**Conclusion:**

Pistachio increased their phytochemical compounds to contrast with abiotic stress. Our data could be useful for introducing special characteristics to the plants, and can be considered when planning a new breeding program or choosing a specific cultivar for a particular use.

## Introduction

Almost 64.5% of Iran's surface area is dry lands, classifying this country among arid countries. In Iran, the average annual rainfall is less than 250 mm. This desert climate provides proper condition for the growth of pistachio (Roostaei, 2004 ▶). Pistachio (*Pistacia vera) *belonging to Anacardiaceae family can tolerate salty and alkaline soils (Tomaino et al., 2010 ▶). Rafsanjan, Damghan, Sarakhs and Feizabad are among the main areas of pistachio cultivation in Iran. Remarkably, native *Pistacia vera *var. sarakhs is used as rootstock (Esfandiyari et al., 2012 ▶). Pistachio kernel is known as a rich source of natural antioxidants, sterols, vitamins, minerals, fatty acids and phenolic compounds. Based on the number and arrangement of carbon atoms, phenolic compounds can be classified as flavonoids and non-flavonoids. Some flavonoids found in pistachio kernel are flavonols, flavanones, isoflavons, flavan-3-ols, proanthocyanidins and anthocyanin. Phenolic acids and stilbenes are non-flavonoid compounds found in pistachio kernel (Brufau et al., 2006 ▶; Hagerman and Butler, 1989 ▶; Miraliakbari and Shahidi, 2008 ▶; Ryan et al., 2006 ▶; Sabaté and Ang, 2009 ▶; Sabaté et al., 2006 ▶; Tomaino et al., 2010 ▶; Tsantili et al., 2011 ▶; Venkatachalam and Sathe, 2006 ▶). Daily use of some valuable compounds might help scavenging free radicals such as reactive oxygen species (ROS). Flavonoids are polyphenol compounds containing fifteen carbons, with two aromatic rings connected via a three- carbon bridge (Corder et al., 2006 ▶). Moreover, flavonoids act as antioxidant through several different mechanisms, including inactivating superoxide and hydroxyl radicals and inhibiting cyclooxygenase, lipooxygenase and xanthine oxidase enzymes (Cos et al., 1998 ▶). 

Proanthocyanidins are a class of polyphenols known as condensed tannins. Their ability in protecting the cells against oxidative damage is higher than that of vitamin C and E. Pistachio is one the dietry sources of proanthocyanidins. It has been demonstrated that the total phenolic and flavonoid contents vary among different cultivars of fruits (Van der Sluis et al., 2001 ▶). 

The effects of Type of cultivars were significant on TPC, TFC, FRAP and DPPH assays results (Tsantili et al., 2011 ▶). DPPH assay is a simple method which is most commonly used for evaluation of the antioxidant capacity of plant extracts. FRAP is also a suitable tool for determining the antioxidant potential. It is based on the reduction of ferric (Fe^3+^) to ferrous (Fe^2+^) ion at low pH, producing a colored ferrous-tripyridyltriazine complex. There is a positive correlation between the total phenolic content and the antioxidant capacity (Gómez-Plaza et al., 2006 ▶; Orak, 2007 ▶). In recent years, the role of some secondary metabolites as protective agents against oxidative damage and the importance of natural antioxidants in many chronic diseases, including cardiovascular disease, type II diabetes and cancers, have been extensively studies (Lopez-Velez et al., 2003 ▶). 

The composition of pistachio kernel may vary depending on the cultivar, cultivation site, climate, and horticultural practice (Agar et al., 1994 ▶; Nadernejad et al., 2012 ▶; Tajabadipour et al., 2005 ▶; Tavallali and Rahemi 2007 ▶; Tsantili et al., 2010 ▶). It is necessary to evaluate the effects of cultivar, rootstock, geographical and climatic condition on some characteristics of agricultural crops, like pistachio, and investigate the effects of these parameters on the amount of fatty acids, mineral elements and total protein in pistachio cultivars (Chahed et al., 2008 ▶; Küçüköner and Yurt, 2003 ▶). The effect of rootstock type on pistachio kernel characteristics has been reported (Tavallali and Rahemi, 2007 ▶). Alterations in moisture levels have been shown in pistachio cultivars collected from different geographical and climatic conditions (Chahed et al., 2008 ▶). According to the literature, the fatty acids content in pistachio cultivars varies among different climate conditions (Agar et al., 1994 ▶). Serious changes in phenolic compounds and antioxidant activity have been shown to be attributed to site of cultivation, climate and some postharvest conditions (Kornsteiner et al., 2006 ▶). Research on pistachio antioxidants has been done on Kerman, Bianca and Bronte cultivars (Ballistreri et al., 2009 ▶; Tavakolipour et al., 2010 ▶; Tomaino et al., 2010 ▶) and even unknown samples (Yang et al., 2009 ▶). The present study aimed to (1) investigate total phenolic (TP), flavonoids (TF) and total antioxidant capacity (TAC) in five selected commercial pistachio cultivars from different geographical regions of Iran, (2) identify the characteristics of selected sites which may affect Iranian pistachios phytochemical properties and (3) evaluate the relationship between two factors and their effects on chemical compositions.

## Materials and Methods

**Plant materials and geographical factors**

**Figure 1:**
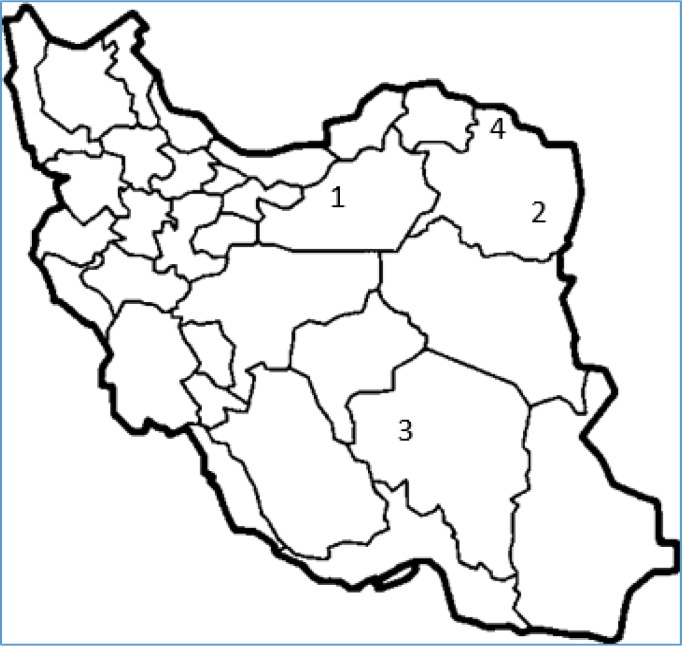
Geographical location characteristics of the cultivation zones. 1: Damghan;,;2: Feizabad,

The ripe fruits of five pistachio (*Pistacia vera*) cultivars including Akbari, Ahmad aghaei, Badami-e-sefid, Kalehghoochi and Owhadi were collected from the four geographical regions of Iran (Damghan, Feizabad, Rafsanjan and Sarakhs) in August and September 2015 (Fig. 1). Kernels and hard shells of fruits were separated and the kernels were air-dried at room temperature. Then, kernels were stored at -18 °C until analysis. Some main characteristics of selected cultivars were described in Table 1. Furthermore, geographical location as well as topographic and climate characteristics of selected sites are shown in Table 4. All chemicals and solvents used in this study were supplied by Merck (Darmstadt, Germany). 

3: Rafsanjan,4: Sarakhs

**Preparation of extracts**

Samples were ground to powder by a mortar and pestle, separately. Thirty grams of each kernel were extracted by 300 ml of 95% methanol for 48 hr at room temperature. Then, the extracts were filtered and evaporated at low pressure, and freeze-dried (OPERON, FDB-5503, Korea). Samples were stored at -80 °C until further assays.

**Determination of total phenolic content (TPC)**

Total phenolic contents were determined using Folin-Ciocalteu method (Heim et al., 2002 ▶). For this purpose, 100 μL of the extract was mixed with 0.5 ml Folin-Ciocalteu reagent (10-times diluted with distilled water). Then, 7 ml of distilled water was added to the solution. After 5 min incubation at room temperature, 1.5 ml sodium bicarbonate (60 mg/ml) solution was added to the mixture and left in the dark for 2 hr. Absorbance was read at 725 nm against blank using UV-visible spectrophotometer (Cecil, UK.). A standard graph was plotted using a standard solution of gallic acid (0.2-1 mg/ml). Results were presented as mg gallic acid/g dry extract (mg GA/g DE). 

**Determination of total flavonoid content (TFC)**

The TFC was measured using a UV-visible spectrophotometer (Cecil, UK.) against a blank sample containing 5 ml extract and 5 ml methanol without AlCl_3_. Absorbance was read at 367 nm (Huang et al., 2004 ▶). Here, 5 ml of 2% aluminum trichloride (AlCl_3_) in methanol was mixed with the same volume of the extract (0.4 mg/ml). Absorbance was read at 367 nm against a blank sample containing 5 ml extract solution with 5 ml methanol without AlCl_3_. The TFC was determined by a standard curve plotted for quercetin. The TFC was expressed as mg of quercetin equivalents (QE)/g dry extract (mg QE/g DE). 

**Determination of total proanthocyanidin content (TPrAC)**

For determination the TPrAC contents, 0.5 ml of supernatant was mixed with 1.5 ml 4% vanillin-methanol solution and 0.75 ml dense HCl. Then, the mixture was incubated at room temperature for 15 min. Next, the absorbance was read at 500 nm against blank using UV-Visible spectrophotometer (Cecil. UK.) (Price et al., 1978 ▶). The TPrAC was expressed as mg of catechin equivalents (CA)/g dry extract (mg CA/g DE). 

**DPPH (2, 2-diphenyl-1-picrylhydrazyl) scavenging capacity assay**

Free radical scavenging activity of the plant extracts was measured by the DPPH method (Fuhrman et al., 2001 ▶). This assay is based on the measurement of the loss of color of DPPH solution as reflected by the change in absorbance at 517 nm due to the reaction of DPPH with the tested sample. The rating of discoloration represents the scavenging potential of different concentrations of the extract. In brief, 0.1 mM solution of DPPH in methanol was prepared and 3 ml of the solution was added to 1 ml of different concentrations (100-300 μg/ml) of the extract. The mixture was shaken strongly for about 10 sec and incubated at room temperature for 60 min. The absorbance was measured at 517 nm using a visible spectrophotometer. Lower absorbance of the reaction mixture demonstrates higher free radical scavenging activity which is calculated using the following equation: 

Percent (%) inhibition of *DPPH activity = ([(A0 – A1 / (A0)] × 100%*

Where A0 is the absorbance of the blank sample and A1 is the absorbance of the test sample. A curve of inhibition percentage or scavenging percentage was plotted against samples concentrations and the concentration of the sample required for 50% inhibition was determined. Accordingly, DPPH assay data was expressed as IC_50_ and percentage of inhibition. Lower IC_50_ value indicates higher antioxidant activity. Butylatedhydroxytoluene (BHT) and vitamin C were used as standard antioxidants. 

**FRAP (Ferric reducing/antioxidant power) assay**

One of the exact and repeatable methods used for determination of the total antioxidant capacity is FRAP assay, which was developed based on the ability of antioxidant compounds to reduce the complex ferric Fe (III) – tripyridyltriazine to ferrous Fe (II) - TPTZ giving a chromatic blue color with the maximum absorbance at 593 nm. The solution (300 mM acetate buffer, 10 mM TPTZ in 40 mM of HCl and 20 mM FeCl_3_.6H_2_O (pH 3.6)) was mixed at a ratio of 10:1:1(v/v), then warmed (at 37 °C) for 5 min. After reading the blank, plant extract or standard solution and water were added to FRAP reagent. The absorbance should be read at 0 and 4 min and differences between these two absorbance (at 593 nm) were detected by UV-Visible spectrophotometer (Cecil. UK.), and compared with the standard curve. Iron (II) sulfate was used as the standard. The assay was carried out in triplicate and the results were reported as mmol of Fe (II)/g dry extract (Razali et al., 2012 ▶). BHT and vitamin C were used as standard antioxidants. 

**Statistical analysis**

Statistical analysis was performed as a factorial complete randomized design with three replications by JMP 8 (SAS Campus Drive, Cary, NC 27513) and Excel software. Significant differences among mean values were determined, by using LSD at the 0.05 level. 

## Results

Two way ANOVA-repeated measures [treatment effect: (cultivar effect: Table 2), (site effect: Table 7), (cultivar × site interaction Table 8), p˂0.05]. 

**Table 1:** Some main characteristics of the five analyzed pistachio cultivars

**Cultivar**	**Nut Shape**	**Size**	**Shell color**	**Most type use**
**Ahmad aghaei**	Jumbo	18-20/20-22/22-24/24-26/26-28/28-30/30-32	cream	Dried fruit
**Akbari**	Jumbo	18-20/20-22/22-24/24-26/26-28/28-30/30-32	Light cream	Dried fruit
**Kalehghoochi**	Round	18-20/20-22/22-24/24-26/26-28/28-30/30-32	cream	Dried fruit
**Owhadi (Fandoghi)**	Round	18-20/20-22/22-24/24-26/26-28/28-30/30-32	cream	Dried fruit
**Badami-e-sefid**	Jumbo	18-20/20-22/22-24/24-26/26-28/28-30/30-32	Light cream	Dried fruit-Fresh

**Table 2:** Cultivar influence on TPC, TFC, TPrAC, IC_50_ and FRAP values in samples collected from the same site^a^

**FRAP** f	**IC50** e	**TPrAC** d	**TFC** c	**TPC** b	**Cultivar**
7.729±0.05^c^	9.181±0.01^c^	132.264±0.4^c^	108.809±0.2^c^	136.637±0. 5^c^	**Ahmad aghaei**
8.265±0.06^a^	8.179±0.05^e^	133.769±0.3^a^	109.518±0.5^a^	137.369±0. 5^a^	**Akbari**
7.864±0.07^b^	8.835±0.03^d^	131.988±0.5^b^	109.346±0.5^b^	136.929±0.1^b^	**Kalehghoochi**
7.344±0.05^e^	9.755±0.05^a^	130.113±0.3^e^	107.584±0.5^e^	135.710±0.5^e^	**Owhadi (Fandoghi)**
7.598±0.05^d^	9.466±0.05^b^	132.431±0.1^d^	105.784±0.5^d^	135.884±0.5^d^	**Badami-e-sefid**

a Means ± SD (standard deviation) in a column not connected by same letter are significantly different at p˂0.05.

b (TPC, mg gallic acid equivalents per g of dried plant);

c (TFC, mg quercetin equivalents per g of dried plant);

d (TPrAC, mg catechin equivalents per g of dried plant);

e DPPH (IC_50_, µg per ml);

f (FRAP, mmol per g)

**Simple effects of cultivar**

**type on TPC, TFC, TPrAC in samples collected from the same site**

As shown in Table 2, there is a significant difference in TPC among selective cultivars collected from different sites. Mean comparison showed significant differences among the cultivars (p˂0.05). Results of the cultivar influence on TPC, TFC, and TPrAC, in samples collected from the same site, showed that among the selected cultivars, Akbari pistachio cultivar with 137.369±0.005 (mg GA/g DE) total phenol, 109.518±0.005 (mg QE/g DE) flavonoids and 133.769 (mg CA/g DE) proanthocyanidins contents, was the richest type in terms of the above-mentioned chemicals. TPC, TFC, and TPrAC content of the other cultivars decreased in the following order: Kalehghoochi, Ahmad aghaei, Badami-e-sefid and Owhadi (Fandoghi). 

**Cultivar influence on antioxidant activity in samples collected from the same site**

The results of BHT and vitamin C antioxidant activity are shown in Table 3. The effects of cultivar type on antioxidant activity are shown in Table 2. Good coefficient (r^2^=0.9834) revealed that there was a positive statistical correlation between TPC, TFC and TPrAC and antioxidant activity (in DPPH and FRAP assays). DPPH and FRAP assays showed similar results. Akbari pistachio cultivar with less IC_50_ (8.179 μg/ml) and great amount of FRAP (8.265±0.00 mmol/g), was the most potent cultivar followed by Kalehghoochi, Ahmad aghaei, Badami-e-sefid and Owhadi (Fandoghi).

**Table 3:** Antioxidant activity of BHT and vitamin C as assessed by DPPH and FRAP assays

**(DPPH)** **IC**_50_** (µg/ml)**	**(FRAP)** **IC**_50_** (µg/ml)**	**Sample**
13.47	15.06	**BHT**
9.17	10.33	**Vit C**

**Simple effects of geographical, topographic and climate characteristics of the selected sites**

All geographical, topographic and climate characteristics of arid and semi-arid selected sites are listed in Table 4. 

**Table 4:** Geographical location, as well as topographic and climate characteristics of the studied zones

**Station**	**Latitude (N)**	**Longitude (E)**	**Altitude (m)**	**Mean annual temperature (’C)**	**Mean annual precipitation (mm)**	**Average of relative humidity (%)**
**Rafsanjan**	30°41´	55°9´	1580.9	15	105	29
**Damghan**	36°04´	54°25´	1180	13	127	55
**Sarakhs**	36°32´	61°10´	235	17.9	189.6	49
**Feizabad**	35°01´	58°78´	940	28.65	20	26

**Table 5:** Physical and chemical properties of the soil collected from different regions

**Station**	**Depth (cm)**	**pH**	**EC (dsm** ^-1^ **)**	**Sand (%)**	**Silt (%)**	**Clay (%)**	**SAR**
**Rafsanjan**	0-30	9.03	21.09	55.1	25.7	19.2	22.40
	30-60	9.04	21.86	81	11	8	33.80
**Damghan**	0-30	7.66	9.44	15.6	52.9	31.5	14.20
	30-60	7.70	11.07	42.1	40.3	17.6	18.3
**Sarakhs **	0-30	8.70	15.12	51	32	17	13.93
	30-60	8.74	17.43	68	24	8	20.87
**Feizabad**	0-30	7.21	4.12	36.10	40.13	23.77	11.06
	30-60	7.23	4.08	56.11	29.78	14.11	13.89

**Physical and chemical properties of soil and water collected from selected sites**

The information about the physical and chemical properties of soil and water collected from different regions are shown in Tables 5 and 6. Soil analysis was conducted in 2 depth (0-30 and 30-60 cm) for all of the samples. The results showed that none of the soil samples had a pH below 7.21 which was related to Feizabad soil sample, while Rafsanjan had the maximum soil EC and SAR (21.86 dsm^-1^, 33.80, respectively). The main common characteristic of all samples collected from different sites was high salinity and alkaline properties. The soil texture properties were reported in Table 5. Also, similar physical and chemical properties of water were presented in Table 6. The maximum levels of pH, EC and SAR of water were identified in samples from Rafsanjan, and Sarakhs, followed by Damghan and Feizabad samples.

**Table 6.** Chemical properties of water samples collected from different regions

**Station**	**pH**	**EC**	**SAR**
**Rafsanjan**	8.88	15.21	27.01
**Damghan**	7.04	6.54	10.03
**Sarakhs **	7.46	12.88	19.42
**Feizabad**	6.98	4.43	4.62

**Effects of region of cultivation on TPC, TFC, TPrAC, in the same cultivar type**

Results showed a significant difference among difference regions (p˂0.05) (Table 7). Pistachio cultivars which were collected from Rafsanjan, showed the highest TPC (154.717 mg GA/ g DE), TFC (128.919 mg QE/g DE) and TPrAC (147.512 mg CA/g DE) and the other sites were in the following order: Sarakhs˃Damghan˃Feizabad. 

**Effects of region of cultivation on antioxidant activity in the same cultivar type**

The results of simple effects of region of cultivation on antioxidant activity are shown in Table 7. A positive correlation was seen between two assays, and among the selected phytochemical parameters and antioxidant activity (r^2^=0.96 and 0.983, respectively). The minimum antioxidant activity of pistachios was seen in samples collected from Feizabad. High values of IC_50_ and FRAP were measured in pistachio cultivars collected from Rafsanjan (8.454 and 9.949, respectively). 

**Table 7:** Effects of region of cultivation on TPC, TFC, TPrAC, IC_50_ and FRAP in the same cultivar^a^

**FRAP** f	**IC50** e	**TPrAC** d	**TFC** c	**TPC** b	**Site**
6.755±0.02^c^	9.158±0.02^b^	128.884±0.3^c^	99.129±0.5^c^	130.133±0 5^c^	**Damghan**
6.21±0.01^d^	9.722±0.02^a^	121.008±0.3^d^	97.520±0.5^d^	129.270±0. 5^d^	**Feizabad**
9.949±0.01^a^	8.545±0.02^d^	147.512±0.5^a^	128.919±0.5^a^	154.717±0.4^a^	**Rafsanjan**
8.127±0.01^b^	8.907±0.02^c^	130.812±0.5^b^	107.265±0.2^b^	131.903±0.5^b^	**Sarakhs**

a Means ± SD (standard deviation) in a column not connected by same letter are significantly different at p˂0.05.

b Total phenolic content (TPC, mg gallic acid equivalents per g of dried plant)

c Total flavonoid content (TFC, mg quercetin equivalents per g of dried plant)

d Proanthocyanidin content (TPrAC, mg catechin equivalents per g of dried plant)

e DPPH radical scavenging activity (IC_50_, µg per ml)

f ferric reducing activity (FRAP, mmol per g)

**Table 8:** Effects of cultivar type × site of cultivation on TPC, TFC, TPrAC, IC_50_ and FRAP^a^

**Station**	**Cultivar**	**TPC** b	**TFC** c	**TPrAC** d	**IC50** e	**FRAP** f
	Ahmad aghaei	129.88±0.5o	98.161±0.6q	128.792±0.5m	8.52±0.07n	6.899±0.09m
	Akbari	130.90±0.5k	99.324±0.6p	129.333±0.5l	8.32±0.07p	7.130±0.09l
**Damghan**	Kalehghoochi	132.02±0.5i	100.903±0.7l	130.398±0.5i	8.029±0.02r	7.146±0.09k
	Owhadi (Fandoghi)	129.156±0.4p	95.412±0.5r	127.989±0.5n	8.796±0.06k	6.419±0.09o
	Badami-e-sefid	128.703±0.4s	93.796±0.5s	127.417±0.5o	9.06±0.04i	6.183±0.06r
	Ahmad aghaei	130.173±0.4m	101.280±0.5k	122.533±0.5p	8.452±0.04o	6.493±0.06n
	Akbari	130.116±0.4n	100.69±0.5m	122.026±0.7q	8.627±0.02l	6.32±0.05p
**Feizabad**	Kalehghoochi	129.043±0.3q	100.443±0.5n	120.876±0.8r	9.047±0.08j	6.196±0.08q
	Owhadi (Fandoghi)	128.876±0.3r	100.053±0.7o	120.733±0.9s	9.172±0.03h	6.12±0.08s
	Badami-e-sefid	128.140±0.3t	93.176±0.7t	118.870±0.7t	9.239±0.05f	5.92±0.09t
	Ahmad aghaei	155.219±0.3^c^	129.158±0.7^c^	147.654±0.7^c^	9.185±0.05g	10.023±0.09^c^
	Akbari	156.42±0.5^a^	130.94±0.6^a^	152.816±0.7^a^	6.457±0.05t	11.49±0.07^a^
**Rafsanjan**	Kalehghoochi	153.864±0.4^d^	127.544±0.6^d^	144.872±0.7^d^	10.732±0.05^c^	9.147±0.09^d^
	Owhadi (Fandoghi)	152.197±0.5^e^	126.813±0.6^e^	140.31±0.7^e^	11.289±0.06^b^	8.70±0.06^e^
	Badami-e-sefid	155.886±0.5^b^	130.141±0.6^b^	151.907±0.7^b^	8.129±0.07q	10.328±0.09^b^
	Ahmad aghaei	131.268±0.5j	106.635±0.6i	130.076±0.7j	10.568±0.05^d^	8.042±0.05i
	Akbari	132.041±0.5h	107.117±0.6h	130.90±0.9h	9.314±0.05^e^	8.122±0.08h
**Sarakhs**	Kalehghoochi	132.79±0.2f	108.492±0.7f	131.804±0.5f	7.532±0.05s	8.429±0.07f
	Owhadi (Fandoghi)	132.612±0.5g	108.058±0.4g	131.421±0.8g	8.607±0.05m	8.135±0.04g
	Badami-e-sefid	130.806±0.3l	106.020±0.6j	129.858±0.8k	12.593±0.09^a^	7.907±0.08j

a Means ± SD (standard deviation) in a column not connected by same letter are significantly different at p˂0.05.

b Total phenolic content (TPC, mg gallic acid equivalents per g of dried plant);

c Total flavonoid content (TFC, mg quercetin equivalents per g of dried plant);

d Proanthocyanidin content (TPrAC, mg catechin equivalents per g of dried plant);

e DPPH radical scavenging activity (IC_50_, µg per ml)

f ferric reducing activity (FRAP, mmol per g)

**Interaction effects among cultivars and sites with phytochemicals parameters and Antioxidant capacity**

There was a significant difference between treatments in interaction effects among cultivars and sites (Table 8). The highest TPC (156.42 mg GA /g DE), TFC (130.94 mg QE/g DE) and TPrAC (152.816 mg CA/g DE) were measured in Akbari cultivar collected from Rafsanjan followed by Badami-e-sefid and Ahmad aghaei. Similar results were observed for DPPH and FRAP assays. A good correlation (r^2^=0.981) was seen between the antioxidant activity and TPC, TFC and TPrAC. On the other hand, the minimum amount of secondary metabolites was seen in Badami-e-sefid from Feizabad region. 

## Discussion

Natural phenolics are potent inhibitors of LDL oxidation and they can decrease the thrombosis damage. Therefore, studying these antioxidative compounds, is favorable. Considerable differences were reported among the type of cultivar, stock, geographical and climate conditions and other factors affecting garden management (Pham-Huy et al., 2008 ▶). The high absorbance value represents the further reducing power. Our results are in agreement with pervious findings that revealed the antioxidant capacity variations among pecan cultivars as measured by DPPH assay (Villarreal-Lozoya et al., 2007 ▶). The amounts of TPC in pecan cultivars, were correlated to DPPH (r^2^= 0.98). Previously, a significant difference in the TPC, TFC contents and antioxidant capacity, among the pistachio cultivars, was shown and it was reported that there is a positive correlation between the TPC and TFC, TFC and DPPH and TFC and FRAP (Tsantili et al., 2010 ▶). The phytochemical evaluation of pistachio cultivars was validated our results that antioxidant capacity and total phenolic contents varied among the pistachio cultivars, and the Akbari pistachio cultivar showed the maximum antioxidant activity (DAVARYNEJAD et al., 2012 ▶) 

The environmental conditions can influence the chemical characteristics of cultivars (Davazdahemami et al., 2011 ▶). Evaluation of plants chemical composition in different countries have shown marked variations (Amiri et al., 2015 ▶; Fattahi et al., 2016 ▶; Rahimmalek et al., 2009 ▶). With regard to cultivar characteristics, it seems that, those with the highest TPC, TFC and TPrAC, appear to be able to withstand environmental stresses such as infertile soil, low quality of water, specific geographical traits and arid and semi-arid climates. Variations in essential oil content of Indian ajowan samples were related to genetics, collection sites, climatic factors, harvesting time and method of extraction (Omer et al., 2014 ▶; Rahimmalek et al., 2009 ▶). In our research, Rafsanjan with high salinity of soil and water, which are stress factors for plant, had the highest content of phytochemical compounds in the pistachio cultivars. We found that this may support the hypothesis that the levels of secondary metabolites of plants increase under stress conditions. 

Our results highlighted that the chemical properties of soil and water as well as genetic factors, affect the quality of pistachio. Understanding and measurement of these differences can be useful in pharmaceutical industry. 

## References

[B1] Agar I, Kaska N, Kafkas S (1994). Characterization of lipids in Pistacia species grown in Turkey. I Int Sym Pistachio.

[B2] Amiri R, Nikbakht A, Etemadi N (2015). Alleviation of drought stress on rose geranium [Pelargonium graveolens (L) Herit] in terms of antioxidant activity and secondary metabolites by mycorrhizal inoculation. Sci Hort.

[B3] Ballistreri G, Arena E, Fallico B (2009). Influence of Ripeness and Drying Process on the Polyphenols and Tocopherols of Pistacia vera L. Molecules.

[B4] Brufau G, Boatella J, Rafecas M (2006). Nuts: source of energy and macronutrients. Br J Nutr.

[B5] Chahed T, Bellila A, Dhifi W (2008). Pistachio (Pistacia vera) seed oil composition: geographic situation and variety effects. Grasas y Aceites.

[B6] Corder R, Mullen W, Khan N (2006). Oenology: red wine procyanidins and vascular health. Nat.

[B7] Cos P, Ying L, Calomme M (1998). Structure-activity relationship and classification of flavonoids as inhibitors of xanthine oxidase and superoxide scavengers. J Nat Prod.

[B8] Davarynejad G, Stefanovits-BaNYAI É, Nagy PT (2012). Investigation of Antioxidant Capacity and Some Bioactive Compounds of Iranian Pistachio (Pistachio vera L) Cultivars. Not Sci Biol.

[B9] Davazdahemami S, Sefidkon F, Jahansooz M, Mazaheri D (2011). Chemical Composition of the Essential Oils from Foliages and Seeds of Ajowan (Trachyspermum ammi (L) Sprague) in two planting dates (spring and summer). J EO Bear Plants.

[B10] Esfandiyari B, Davarynejad G, Shahriari F, Kiani M, Mathe A (2012). Data to the sex determination in pistacia species using molecular markers. Euphytica.

[B11] Fattahi B, Nazeri V, Kalantari S, Bonfill M, Fattahi M (2016). Essential oil variation in wild-growing populations of Salvia reuterana Boiss collected from Iran: Using GC–MS and multivariate analysis. Ind Crops Prod.

[B12] Fuhrman B, Volkova N, Suraski A, Aviram M (2001). White wine with red wine-like properties: increased extraction of grape skin polyphenols improves the antioxidant capacity of the derived white wine. J Agri Food Chem.

[B13] Gee GW, Bauder JW, Klute A (1986). Particle-size analysis. Methods of soil analysis Part 1 Phys min methods.

[B14] Gómez-Plaza E, Miñano A, López-Roca JM (2006). Comparison of chromatic properties, stability and antioxidant capacity of anthocyanin-based aqueous extracts from grape pomace obtained from different vinification methods. Food Chem.

[B15] Hagerman AE, Butler LG (1989). Choosing appropriate methods and standards for assaying tannin. J Chem Ecol.

[B16] Heim KE, Tagliaferro AR, Bobilya DJ (2002). Flavonoid antioxidants: chemistry, metabolism and structure-activity relationships. J Nutr Biochem.

[B17] Huang D-J, Chun-Der L, Hsien-Jung C, Yaw-Huei L (2004). Antioxidant and antiproliferative activities of sweet potato (Ipomoea batatas [L] LamTainong 57') constituents. Botan Bull Acad Sin.

[B18] Kornsteiner M, Wagner K-H, Elmadfa I (2006). Tocopherols and total phenolics in 10 different nut types. Food Chem.

[B19] Küçüköner E, Yurt B (2003). Some chemical characteristics of Pistacia vera varieties produced in Turkey. Eur Food Res Tech.

[B20] Lopez-Velez M, Martinez-Martinez F, Valle-Ribes CD (2003). The study of phenolic compounds as natural antioxidants in wine.

[B21] Miraliakbari H, Shahidi F (2008). Lipid class compositions, tocopherols and sterols of tree nut oils extracted with different solvents. J Food Lipids.

[B22] Nadernejad N, Ahmadimoghadam A, Hossyinifard J, Poorseyedi S (2012). Phenylalanin ammonia-Lyase activity, total phenolic and flavonoid contents in flowers, leaves, hulls and kernels of three pistachio (Pistacia vera L) cultivars. American-Eurasian J Agri Environ Sci..

[B23] Omer E, Said-Al Ahl H, El Gendy A (2014). Yield and Essential Oil of Ajwain (Trachyspermum ammi) Plant Cultivated in Saline Soil of North Sinai in Egypt. J EO Bear Plants.

[B24] Orak HH (2007). Total antioxidant activities, phenolics, anthocyanins, polyphenoloxidase activities of selected red grape cultivars and their correlations. Sci Hort.

[B25] Pham-Huy LA, He H, Pham-Huy C (2008). Free radicals, antioxidants in disease and health. Int J Bio Sci.

[B26] Price ML, Van Scoyoc S, Butler LG (1978). A critical evaluation of the vanillin reaction as an assay for tannin in sorghum grain. J Agri Food Chem.

[B27] Rahimmalek M, Tabatabaei BES, Etemadi N, Goli SAH, Arzani A, Zeinali H (2009). Essential oil variation among and within six Achillea species transferred from different ecological regions in Iran to the field conditions. Ind Crops Prod.

[B28] Razali N, Mat-Junit S, Abdul-Muthalib AF, Subramaniam S, Abdul-Aziz A (2012). Effects of various solvents on the extraction of antioxidant phenolics from the leaves, seeds, veins and skins of Tamarindus indica L. Food Chem.

[B29] Roostaei N (2004). River Basin Challenges and Management in Iran.

[B30] Ryan E, Galvin K, O'Connor T, Maguire A, O'Brien N (2006). Fatty acid profile, tocopherol, squalene and phytosterol content of Brazil, pecan, pine, pistachio and cashew nuts. Int J Food Sci Nutr.

[B31] Sabaté J, Ros E, Salas-Salvadó J (2006). Nuts: nutrition and health outcomes. Br J Nutr.

[B32] Sabaté J, Ang Y (2009). Nuts and health outcomes: new epidemiologic evidence. Am J Clinical Nutr.

[B33] Tajabadipour A, Panahi B, Zadehparizi R (2005). The effects of rootstock and scion on early splitting and cracked hull of pistachio. IV Int Sym Pistachios Almonds.

[B34] Tavakolipour H, Armin M, Kalbasi-Ashtari A (2010). Storage stability of Kerman pistachio nuts (Pistacia vera L). Int J Food Eng.

[B35] Tavallali V, Rahemi M (2007). Effect of rootstock on nutrient acquisition by leaf, kernel and quality of pistachio (Pistacia vera L). Am-Eur J Agri Environ Sci.

[B36] Tomaino A, Martorana M, Arcoraci T, Monteleone D, Giovinazzo C, Saija A (2010). Antioxidant activity and phenolic profile of pistachio (Pistacia vera L, variety Bronte) seeds and skins. Biochem.

[B37] Tsantili E, Konstantinidis K, Christopoulos M, Roussos P (2011). Total phenolics and flavonoids and total antioxidant capacity in pistachio (Pistachia vera L) nuts in relation to cultivars and storage conditions. Sci Hort.

[B38] Tsantili E, Takidelli C, Christopoulos M, Lambrinea E, Rouskas D, Roussos P (2010). Physical, compositional and sensory differences in nuts among pistachio (Pistachia vera L) varieties. Sci Hort.

[B39] van der Sluis AA, Dekker M, de Jager A, Jongen WM (2001). Activity and concentration of polyphenolic antioxidants in apple: effect of cultivar, harvest year, and storage conditions. J Agri Food Chem.

[B40] Venkatachalam M, Sathe SK (2006). Chemical composition of selected edible nut seeds. J Agri Food Chem.

[B41] Villarreal-Lozoya JE, Lombardini L, Cisneros-Zevallos L (2007). Phytochemical constituents and antioxidant capacity of different pecan [Carya illinoinensis (Wangenh) K. Koch] cultivars. Food Chem.

[B42] Yang J, Liu RH, Halim L (2009). Antioxidant and antiproliferative activities of common edible nut seeds. LWT-Food Sci Tech.

